# Breast Magnetic Resonance Imaging and Its Impact on the Surgical Treatment of Breast Cancer

**DOI:** 10.1155/2014/632074

**Published:** 2014-04-22

**Authors:** Georgia Tsina, Philippe Simon

**Affiliations:** Hôpital Erasme, Université Libre de Bruxelles (ULB), Belgium Route de Lennik Lennikse Baan 808, 1070 Anderlecht, Belgium

## Abstract

Breast MRI focuses on the detection of multifocality, multicentricity, and bilaterality of newly diagnosed breast cancer. A retrospective study was carried out on 833 patients that were diagnosed and treated for breast cancer between January 2002 and December 2011. Patients were divided into two groups: those that had a presurgery breast MRI and those that did not. The two groups were compared on the basis of the several parameters. The aim of the study was to determine whether the use of MRI in breast cancer screening changes the initial treatment decision. In 18% of the patients, MRI revealed a multifocal or a multicentric unilateral breast cancer, a bilateral tumour, or a larger cancer than initially diagnosed. Most of these patients underwent a second-look breast ultrasound, with or without an additional biopsy. The percentage of mastectomies did not increase as a result of an MRI exam. Neoadjuvant chemotherapy was used more often and the percentage of reoperations decreased when an MRI was performed.

## 1. Introduction

Mammography is the reference exam for the screening and diagnosis of breast cancer. It presents a sensibility of 40–73% and a specificity of 94% that is highly dependent on breast density [1].

Breast ultrasound is complementary to the mammography technique. The combination of the two screening tests offers a sensibility of 92% and specificity of 96% [2].

Breast magnetic resonance imaging (MRI) adds another 3.1% to the total sensibility of the gold standard screening tests, that is, mammography and ultrasound. MRI presents a sensitivity of 91% and a specificity of 88% [1–4].

There are several indications that are often considered before undertaking a breast MRI: evaluation of breast implants [1, 5], any disagreement between the results of the mammography and the ultrasound [5–7], high-risk women, presence of a lobular invasive carcinoma [8–10], follow-up procedure after neoadjuvant chemotherapy, and cases of breast cancer extended to the thoracic wall.

The results of studies published during the last ten years concerning breast MRIs and their correlation with breast cancer diagnosis and treatment are conflicting, and the benefits of using MRI for breast cancer diagnosis have yet to be established.

Two studies of 969 patients [3, 11] showed that an MRI exam can improve the detection of contralateral breast cancer when added to a thorough clinical breast examination and mammographic evaluation at the time of the initial diagnosis. Thirty patients were found to have contralateral breast cancer (3% of patients). MRI presented a sensitivity of 91%, a high negative predictive value at 99%, and a specificity of 88%.

Perono et al. [12] conducted a retrospective study with 525 patients using MRI on women diagnosed with breast cancer. The team found more extensive disease in 27.4% of the patients, which led to a change in the surgical decision in 22.5% of the women. The new surgical decision benefited 18.8% of the women in this study. The overall false positive rate was 27.1%.

On the other hand, Young et al. argued that breast MRI confers no diagnostic advantage over the gold standard tests for early-stage breast cancer and may even lead to worsened patient outcomes [13].

The percentage of mastectomies was decreased in the MRI group of patients in the study of García-Lallana et al. but increased in the study of Miller et al. [14, 15].

This study was designed to determine whether routine breast MRI improves basic screening tests (i.e., mammography and ultrasound) in detecting multicentric or bilateral disease and whether its results have a positive impact on the treatment aptitude and surgical decision.

## 2. Materials and Methods

A retrospective analysis of data on women diagnosed with breast cancer and treated at the Erasme Hospital (Free University of Brussels) between January 2002 and December 2011 was performed.

The Ethics Committee of the Erasme Hospital gave its approval to this project and issued an exemption from obtaining informed consent from the patients.

All patients less than 85 years of age with a positive biopsy for breast cancer obtained at the Erasme Hospital were included. Patients with claustrophobia, a history of breast cancer, stage IV or generalized tumours, genetic mutation of BRCA-1 or BRCA-2, inflammatory breast cancer, or Paget's disease of the nipple were excluded. Women whose breast cancer diagnosis was not obtained at the Erasme Hospital and patients who had been treated at another hospital were also excluded from the study.

Data for 1130 patients were analysed: 297 patients were excluded, leaving a sample of 833 patients for the study.

Patients were categorised into the following two groups:

- Group A: patients without a breast MRI during their presurgery investigation (*N* = 351),
- Group B: patients with a breast MRI during their presurgery investigation (*N* = 482).

The clinical and pathological staging was carried out using the TNM (tumour, node, and metastases) classification (1989).

The MRI results were interpreted as follows:

1. group “idem” when the MRI results were similar to those of the screening tests (mammography and/or ultrasound),
2. group “multifocal” when undiagnosed additional, unilateral tumours (unifocal or multifocal) were discovered,
3. group “bilateral” when an extra bilateral tumour was revealed,
4. group “suspicious” when the MRI modified the BIRAD (Breast Imaging-Reporting and Data System) classification from BIRAD 3 to worse,
5. group “size” when the cancer size estimation was significantly different between the classic screening tests and MRI,
6. group “noncontributory” when the MRI was noncontributory and revealed false negative results, in contrast to the mammography and/or breast ultrasound results.

Group B (i.e., patients that had an MRI) was further divided into two subgroups, I and II. Subgroup I consisted of patients where the initial choice of treatment was not changed as a result of the MRI. Subgroup II consisted of patients where the treatment decision was modified because of the MRI results (i.e., enlarged conservative resection, mastectomy, or neoadjuvant chemotherapy). Subgroup II also included patients who had a bilateral surgery and those whose diagnosis was based on MRI results after receiving a false negative from the conventional tests.

Subgroup II was also studied separately for the following parameters: type of operation, stage and type of cancer, decision to have a second-look ultrasound, decision to have a secondary biopsy, and the results of the MRI exam.

This study aimed to investigate the impact of the MRI on treatment decisions for breast cancer in one centre over a period of ten years.

The statistical software SAS 9.3 was used to analyse the dataset. The relevant frequency tables were generated to describe the data. In addition to SAS 9.3, the statistical software R was used to obtain the relevant plots and regression lines. The key binary variable was “MRI,” which denoted whether a patient underwent a breast MRI. A value equal to 1 was assigned to patients who had undergone an MRI, and a value equal to 0 was assigned to those who did not. Similar binary values were assigned to other variables, such as receiving a second-look ultrasound, an additional biopsy, and a change in the initial treatment plan, where a value equal to 1 was assigned when they did take place and a value equal to 0 when they did not.

## 3. Results

The rate of MRI has risen through the years. Except for 2010 (48%), the use of breast MRI has increased an average of 58% during the period 2006–2011. No significant differences in the occurrence of pathological types of cancer explain the lower use of MRI in 2010.

The MRI results are classified under the six headings presented in Section 2. In almost 20–25% of the patients who underwent a breast MRI, there was a difference between the MRI results and the results of the classic screening tests. In 34.5% of the patients whose MRI results differed from the initial screening test diagnosis, the MRI exam diagnosed a simultaneous unilateral tumour. In 10.6% of the patients, the MRI screening revealed a bilateral cancer, whereas for 15.6% of the patients the tumour was discovered to be significantly larger than originally diagnosed. In 17.7% of these cases, the MRI screening confirmed the presence of cancer despite an ACR3 (American College of Radiology) classification by mammography or ultrasound for the same lesion. It is noteworthy that, in 22% of the cases, the MRI results were noncontributory (false negative results), in contrast to the positive results from the classic screening tests.

Further analysis of the noncontributory MRI cases showed that half of these patients had an invasive ductal carcinoma and the other half an invasive lobular carcinoma (20%) or an* in situ* carcinoma (28%). The average age of these patients was 49 years.

We analysed the incidence of different pathological cancers in the whole study population and separately for Group B, and we compared these with the pathological types in the noncontributory category.

The* in situ *carcinomas represented 18.8% of the whole population, 14.3% of the Group B population, and 28.8% of the noncontributory cases. Lobular carcinomas represented 12.4% of the whole population, 16.6% of the Group B population, and 20% of the noncontributory cases. Finally, invasive ductal carcinomas represented 70% of the whole study and Group B population and half of the noncontributory cases.

**Figure 1:**
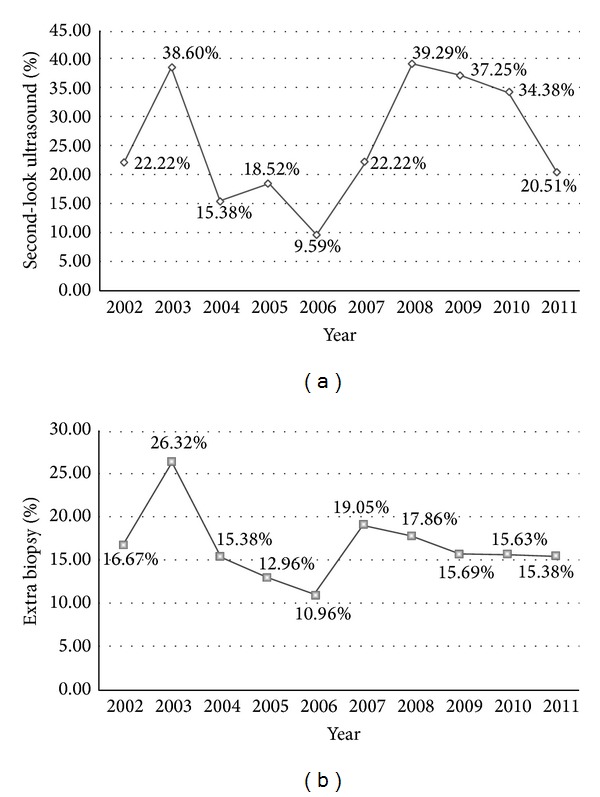
(a) Percentage of second-look ultrasound examination referrals in Group B, per year, throughout the reference period. (b) Percentage of additional biopsy referrals in Group B, per year, throughout the reference period.

As shown in Figures 1(a) and 1(b), an average of 25% of the women that underwent an MRI had to repeat a breast ultrasound, while more than half were recommended to have an additional biopsy.

In our study, 61.6% of Group A patients had a lumpectomy or quadrantectomy, 26.9% a mastectomy, 6.3% underwent neoadjuvant chemotherapy, and 5.16% had a second operation. With regard to Group B patients, 54.8% benefited from a breast-conserving treatment, 25.9% had a mastectomy, 16.6% received neoadjuvant chemotherapy followed by a surgery, and 2.7% required a second operation.

Table 1 presents the frequency per year of cases in which the initial choice of treatment was changed following the MRI results (subgroup II), including the cases where a surgeon performed a large quadrantectomy instead of a simple lumpectomy, performed a mastectomy instead of a breast-conserving treatment, proceeded first to a neoadjuvant chemotherapy before a surgery, or carried out a bilateral surgery.

On average, 10–20% of patients who underwent an MRI examination had their treatment plan modified, and it was more aggressive than the one originally planned after the mammography and breast ultrasound results.

According to these results, almost half of subgroup II patients underwent a nonconserving treatment: 35% had breast-conserving surgery, 11% benefited from neoadjuvant chemotherapy, and approximately 5% required a second surgery as a result of the presence of positive margins after the breast-conserving treatment. Almost half of these patients had a stage I cancer (48%), 26.5% of the patients were in clinical stage II, 7% were in stage III, and 18% had an* in situ* carcinoma. Most of the patients had an invasive ductal carcinoma (51.8%) or an invasive lobular carcinoma (30%). Finally, 74.7% of these patients had a second-look ultrasound and an additional biopsy before their surgery.  Table 2 presents in detail the occurrence of indications for conducting a breast MRI in subgroup II patients.

Around 2/3 of the patients had an indication that justified the decision to perform a breast MRI, such as an invasive lobular carcinoma, a doubtful or contradictory screening result, or a young age. In 37.4% of the cases, there was no particular indication for performing a breast MRI; it was conducted based on a surgeon's decision. This 37.4% of patients represent 6.4% of the Group B population and 3.7% of the whole study population.

When the medical files of the patients with a “no specific reason” MRI indication were further analysed, we did not notice any particularity to their medical, surgical, or family history. The MRI was performed based on the surgeon's decision. We admit that most of these women were patients of the same surgeons.

With statistical analysis, different approaches can be taken to describe the relationships between different measurements. In this study, tests on the frequency tables for the key variables (mammography, ultrasound, MRI, second-look ultrasound, biopsy, and treatment) yielded significant *P* values (smaller than the limit of 0.05). This indicates that these variables are somehow associated with the variable treatment. According to our statistical analysis, our model is 99.52% accurate.

## 4. Discussion

Breast MRI has been increasingly used in the last five years, confirming the general tendency for breast MRI as a routine presurgery procedure.

This study constituted a retrospective, observational report of patients with a newly confirmed diagnosis of breast cancer. As a retrospective study, it has certain limitations. In our study, the first bias is that the population of our study has, by definition, breast cancer. Therefore, it is difficult to assess the benefit of the breast MRI, since we excluded the cases where classical screening test results presented a false positive and MRI results were negative for a breast malignancy. Secondly, while the sample size was sufficiently large to make the results statistically reliable, there is no follow-up, which makes it difficult to perform a conclusive statistical analysis.

The results from breast MRI are similar to the conventional screening tests in 76.6% of the cases. In 15% of the cases, additional tumours were diagnosed based on MRI screening (8% an extra unilateral tumour, 2.5% an extra bilateral tumour, and 3.5% the tumour size was larger than initially diagnosed). In 4.1% of the patients, MRI discovered the presence of a cancer different to the initial screening tests. In 5.18% of the cases, MRI was noncontributory (52% with an invasive ductal cancer, 20% with a lobular carcinoma, and 28% with an* in situ *cancer).

On average, more than a quarter of patients who underwent a breast MRI had a second-look breast ultrasound. An extra biopsy was performed in 50% of them. Many contralateral surgeries and mastectomies could have been avoided if a needle biopsy had been performed before the surgery [16]. The percentages revealed by this study are in agreement with the results of other recent studies. In a study by Calvo-Plaza et al. [17], MRI detected an additional breast disease in 39% of the cases (98 patients studied), requiring an additional biopsy in 20% of these patients. In a study by Grady et al. [20], 14% of patients received an additional biopsy after MRI (compared to 16% in this study).

The comparison of the types of treatments between Group A and Group B revealed a higher percentage of presurgery neoadjuvant chemotherapy after MRI (16.60% versus 6.30%, resp.) and a higher percentage of repeated surgery in Group A (5.16% versus 2.7%, resp.). There was no increase in breast nonconserving treatment in the MRI group. The percentage of mastectomies between the two groups was similar (around 26%). Breast-conserving treatment was used more often (61.6%) in non-MRI patients than in Group B (54.77%).

Almost 50% of the patients that underwent an MRI had a more aggressive treatment than those that did not (a quadrantectomy, mastectomy, or neoadjuvant chemotherapy).

In articles published recently, the benefit of a routine presurgery breast MRI was put into question. In a study of 3112 patients [18], MRI was demonstrated to be responsible for an increased percentage of mastectomies and an unfavourable harm-benefit balance in the choice of treatment. By contrast, the studies of Pediconi et al. [19] and Grady et al. [20] demonstrated that MRI positively affected patient management and was recommended for mapping tumour size in patients newly diagnosed with breast cancer. Mastectomy percentages did not increase because additional confirmatory biopsies were performed before making treatment decisions.

In this study, an average of 17.2% of patients each year received a beneficial change in their treatment plan after a presurgery breast MRI. Similarly, in a study by Perono et al. [12], an additional malignant lesion was detected in about one in every five patients who underwent a breast MRI, which resulted in a beneficial modification to the surgical treatment plan in 18.8% of these patients because of the additional biopsies that were carried out as a follow-up to the MRI results.

Examination of the indications for a breast MRI exam in subgroup II revealed that the majority of patients had a doubtful initial screening test (36%), which is why they were referred for an MRI. From this group, 23% of patients had a lobular invasive carcinoma and 3.6% were young women (less than 40 years old). Nevertheless, 37.4% of patients underwent an MRI, and as a result, they were submitted to a more aggressive treatment, which was justified afterwards by the pathology results. However, initially there was no clear indication for performing an additional MRI. This 37.4% of patients in subgroup II represent 6.4% of the population of Group B and 3.7% of the whole study population. Therefore, if a presurgery breast MRI is carried out in 100 non-high-risk women with newly diagnosed breast cancer, 3-4 of them may be diagnosed with an additional unilateral (61.3%) or bilateral (9.6%) tumour, a larger tumour (16.1%), or a suspicious lesion (13%). The patients whose additional bilateral tumour was discovered by accident represented 0.36% of the study population, while patients with an additional unilateral tumour represented 2% of the population.

Is it then worthy to carry out a routine MRI to all patients with a newly diagnosed breast cancer, even if they fall under no official indications for this screening? Is there a cost- effectiveness of a routine presurgery MRI, when in 1000 MRIs 3 bilateral cancers and 20 extra unilateral foci were revealed? Many of the additional diagnosed tumours would regress after systemic treatments in many cases (chemotherapy, hormonotherapy, and radiotherapy), and routine breast MRI will likely have little impact on patient survival rates [21].

## 5. Conclusions

Breast MRI is a useful technological innovation, provided that the newly discovered lesions are confirmed by biopsies prior to surgery.

Neoadjuvant chemotherapy is used more often when breast MRI is performed, which likely accounts for the decrease in repeated surgery when breast MRI is used for breast cancer diagnosis.

However, the results suggest that breast MRI should be undertaken when patients fulfil a specific criteria and not as a routine presurgery exam.

Undoubtedly, the detection of additional lesions using breast MRI makes it compulsory to perform a second-look ultrasound, coupled with an additional biopsy, in order to reduce the risk of unjustified nonconserving surgery.

## Figures and Tables

**Table 1:** Frequencies and rates of subgroup II patients per year.

Frequencies and rates of subgroup II patients per year			
Year	*n*	*N*	*P*
2002	3	18	16.67%
2003	16	57	28.07%
2004	8	39	20.51%
2005	7	54	12.96%
2006	7	73	9.56%
2007	11	63	17.46%
2008	11	56	19.64%
2009	9	51	17.65%
2010	5	32	15.63%
2011	6	39	15.38%
Total	**83**	**482**	**17.22%**

*n*: the number of patients from subgroup II per year; *N*: the number of patients in Group B per year; *P*: the percentage of “*n*” each year.

**Table 2:** Percentage of MRI indications in patients in subgroup II.

Indication	Number of patients	Percentage
Invasive lobular carcinoma	19	22.9%
Doubtful or contradictory screening test results	30	36.1%
Age < 38 years	3	3.6%
No specific reason	31	37.4%
Total	**83**	**100%**
